# Exploring and understanding HCV patient journeys- HEPCARE Europe project

**DOI:** 10.1186/s12879-021-05928-9

**Published:** 2021-03-05

**Authors:** Shannon Glaspy, Gordana Avramovic, Tina McHugh, Cristiana Oprea, Julian Surey, Irina Ianache, Juan Macías, Alistair Story, Walter Cullen, John S. Lambert

**Affiliations:** 1 School of Medicine, University College Dublin, Dublin, Ireland; 2 Mater Misericordiae University Hospital, Dublin, Ireland; 3 Victor Babes Clinical Hospital for Infectious and Tropical Diseases, Bucharest, Romania; 4 University College London, London, UK; 5 Universidad Autónoma Madrid ES, Madrid, Spain; 6 Hospital Universitario de Valme, Sevilla, Spain; 7 University College London Hospital, London, UK; 8 Consultant in Infectious Diseases, Medicine and Sexual Health, Mater Misericordiae University Hospital, Dublin 7, Ireland

**Keywords:** Hepatitis C, Vulnerable populations, People who inject drugs (PWID), Homeless, Lost to follow up (LTFU), Integrated HCV care

## Abstract

**Background:**

Hepatitis C Virus (HCV) is a leading cause for chronic liver diseases worldwide. The European Union and World Health Organization aspire to eliminate HCV by 2030. However, among at-risk populations, including, homeless people, prisoners and People Who Inject Drugs, access to diagnosis and treatment is challenging. Hepcare Europe is an integrated model of care developed to address this by assessing potential reasons for these restrictions and determining measures needed to improve HCV diagnosis, treatment and access to care within different communities.

**Objectives:**

HepCare Europe is an EU-supported project involving collaboration between five institutions in: Ireland, United Kingdom, Spain and Romania. We aim to explore the journey of care experienced by those living with HCV with a focus on previous care disruptions (loss to follow up) and the new HepCare Europe Programme.

**Methods:**

Research teams conducted semi-structured interviews with patients who accessed services through HepCare Europe thus, patients were recruited by purposeful sampling. Patients interviewed had received, or were in the final weeks of receiving, treatment. The interviews were audio recorded, transcribed and translated into English, and sent to the Dublin team for inductive thematic analysis. Researchers from the HepCare Europe research team coded the data separately, then together.

**Results:**

Common themes are introduced to present similarities, following individual site themes to highlight the importance of tailored interventions for each country. Key themes are: 1) Hepatitis C patients lost to follow up 2) HepCare improved access to treatment and 3) the need for improved HCV education. Individual themes also emerged for each site. These are: Ireland: New opportunities associated with achieving Sustained Virologic Responses (SVR). Romania: HCV is comparatively less crucial in light of Human Immunodeficiency Viruses (HIV) coinfections. UK: Patients desire support to overcome social barriers and Spain: Improved awareness of HCV, treatment and alcohol use.

**Conclusion:**

This study identified how the tailored HepCare interventions enabled improved HCV testing and linkage to care outcomes for these patients. Tailored interventions that targeted the needs of patients, increased the acceptability and success of treatment by patients. HepCare demonstrated the need for flexibility in treatment delivery, and provided additional supports to keep patients engaged and educated on new treatment therapies.

## Background

Hepatitis C Virus (HCV) is a major cause of liver disease worldwide [1]. It is estimated that 71 million people are chronically infected with the HCV virus [2] with approximately 5.6 million within the European Union (EU). The long-term consequence of HCV infection is severe, as chronic infections are associated with liver cirrhosis and hepatocellular carcinoma [3]. The World Health Organisation’s (WHO) global health strategy on viral hepatitis is determined to eliminate viral hepatitis as a global health threat by 2030 [4].

People who inject drugs (PWID) and people with a history of injecting drug use are highly equated with HCV infection in Europe and are a cohort associated with new infections of the virus, with estimates indicating that 1.2 million PWID in Europe have been HCV infected [5]. Additionally, members of the prison population and homeless people require additional care in the EU. It is estimated that approximately half of all prison members have a history of illicit drug use [6], while studies have shown that the highest absolute rate of disease in homeless individuals is for hepatitis C [7, 8]. To achieve HCV elimination, it is essential to acknowledge the most vulnerable populations, including prisoners, homeless persons and PWIDs for both research and clinical care. Reaching these populations requires new strategies, including simplifying the cascade of care and involving partnerships with HCV specialists and community healthcare providers [9, 10].

A recent study notes that HepCare Europe, an integrated model of care, improved access to treatment by targeting vulnerable populations and linking them to care. The study reports that, a major outcome of this model of care was that a total of 2608 participants were recruited across 218 sites in four European cities. HCV antibody test results were obtained for 2568 (98.5%); 1074 (41.8%) were antibody-positive, 687 (60.5%) tested positive for HCV-RNA, 650(60.5%) were linked to care, and 319 (43.5%) began treatment. 196 (61.4%) of treatment initiates achieved a Sustained Viral Response (SVR) at dataset closure,108 (33.9%) were still on treatment, eight (2.7%) defaulted from treatment, and seven (2.6%) had virologic failure or died [11].

While a plethora of both quantitative and qualitative literature exists on pre-Direct Acting Antiviral (DAA) HCV treatment, there is sparse literature for this new era of HCV treatment. Recent developments in treatment offer cure rates > 90%. However, the potential of these treatments will only be realised if HCV identification among PWID with linkage to treatment is optimised and therefore requires research investigating new interventions [9]. DAA’s are less invasive, present less risks to one’s health, and are less costly and more effective than previous interferon treatment. Despite this, the fear of interferon treatments remains amongst patients due to a lack of awareness of new therapies [12–14], presenting an additional hurdle to the list of well-documents barriers to engaging vulnerable populations in HCV care [12, 15]. In light of new advancements in medication but also in screening and assessment, new initiatives accommodating complex lifestyles have become possible including increased nurse and peer support [12, 13, 16–19]. However, many of these studies come from the UK and currently there are no studies that engage multiple cohorts across Europe.

HepCare Europe is an EU funded project with sites in four countries: Ireland, UK, Romania and Spain. The project aims to develop, implement and evaluate interventions to improve HCV diagnosis, evaluation and treatment among vulnerable populations. The structure of the HepCare intervention allowed for each site to tailor the testing and treatment plan to the needs of their populations, providing peer support and community and prison-based treatment where appropriate [20]. HepCare focuses on screening and identifying new and previously known HCV positive cases and linking them to care. In this study we aim to identify the reasons why this patient population are lost to follow up when seeking access to services and how to keep them engaged in care through interviews with patients who have completed treatment.

## Methods

### Ethics

Ethical approval was granted by the Institutional Review Boards in each of the following sites: Mater Misericordiae University Hospital (Dublin, Ireland); North-West Haydock Research Ethics Committee (London, UK); Hospital Universiario de Valme (Seville, Spain); and Victor Babes Clinical Hospital for Infectious and Tropical Diseases (Bucharest, Romania). Surveillance and supervision for the study were provided through the governance framework of the HepCare Europe Project. All methods were carried out in accordance with relevant guidelines and regulations.

### Study design

This study adopted a qualitative approach to gain a thorough understanding of reasons for loss to follow-up in receiving HCV treatment. Utilizing semi-structured interviews meant the researchers could obtain a first-hand apprehension of the circumstances that hinder a patient’s access to treatment. Inductive thematic analysis was used following the development of major and minor themes during the data analysis stage. Researchers held semi-structured interviews with patients in person and ten interviews were selected for each site to ensure equal representation across the four countries. Due to voluntary participation, participants were not reimbursed.

Key areas for exploration were agreed and disseminated to each site. This allowed for sites to tailor questions to location, population and differences in intervention procedures and administration. The topics included HCV journey, barriers to treatment, addiction issues, effects of treatment, the treatment programme and peer experience.

### Settings and recruitment

The study was conducted across four European countries; Ireland (Dublin, Cork), UK (London), Spain (Seville), and Romania (Bucharest). The inclusion criteria included the capacity to provide informed consent, being positive for HCV infection and having initiated treatment under the Hepcare initiative. Purposeful sampling was used to identify 10 patients from each country (*n* = 40) that were representative of the greater site cohort’s characteristics (see Table 1 ‘Recruitment’). Despite attempts to achieve the desired representation, it was not possible to obtain participants that matched all desired requirements because 1) patients had left the service (prison, etc), 2) patients declining invitation to interview, 3) patients were unable to be contacted or patients had not yet completed treatment (Lost to follow up). Informed consent was obtained from all participants involved in this study prior to the data collection stages.

**Table 1:** Recruitment

Country	Ireland				UK				Spain				Romania			
	Desired		Actual		Desired		Actual		Desired		Actual		Desired		Actual	
Men	10		10		8		7		9		9		8		8	
Women	0		0		2		3		1		1		2		2	
Age	< 25	1	< 25	0	< 25	0	< 25	0	< 25	0	< 25	0	< 25	1	< 25	1
	25–34	3	25–34	1	25–34	1	25–34	1	25–34	1	25–34	0	25–34	4	25–34	3
											35–54	7	35–54	5	35–54	6
	35–54	6	35–54	8	35–54	7	35–54	7	35–54	7	> 55	3	> 55	0	> 55	0
	> 55		> 55	1	> 55	2	> 55	2	> 55	2						
IDU Ever	8		10		9		9		8		8		9		10	
Prev LTFU	4		10		9		8		6		8		1		9	
New Cases	6		0		1		2		4		2		9		1	

### Data collection

Ten semi-structured interviews were collected in Bucharest, Romania, 10 interviews in Seville, Spain, 10 interviews in the UK and 10 interviews from Mountjoy Prison in Dublin, Ireland during the 2nd and 3rd year of the HepCare Europe Project. Conducting 10 interviews in each country meant that equal representation was secured across all four sites, which allowed for a fairer analysis of the data generated from the interviews. Interviews were audio recorded and transcribed by the researchers. They were translated at each site into English and sent to Dublin for analysis. Interviews were tailored to discuss the specific interventions used in each site. In Ireland, the intervention took place in a prison setting, therefore participants were interviewed about the acceptability of receiving HCV treatment in the prison setting. In London, participants were identified through homeless outreach services and community interventions. In Romania, patients were recruited through prisons, homeless shelters, addiction services and a hospital outpatient clinic. In Spain, people who inject drugs (PWIDs) were recruited from homeless and addiction services.

### Data analysis

Two rounds of inductive thematic analysis were conducted the first round was conducted by the fourth author and the second round was conducted by the second and third author. A small set of pre-defined codes were used in conjunction with emerging codes. Researchers coded the interviews separately and then together, grouping codes into categories and classifying them into the identified themes. See Table 2 for ‘Example of Analysis Process’. As the interventions at each site were similar but not identical, interviews were analysed together by site. After identifying the themes from each site, the site results were then compared and the overarching themes were identified, along with some themes that were unique to the sites. Through this process, two main themes and one minor theme were identified. The major themes: 1) Hepatitis C patients had been lost to follow up 2) HepCare improved access to treatment, and the minor theme: 3) The need for widespread improved HCV education (public, patients, prisoners, and health care providers). See Table 3 for ‘Theme Progression’. Additional themes identified from individual sites are as follows: Ireland: Achieving Sustained Virologic Responses (SVR) associated with new opportunities, Romania: HCV comparatively less important in light of Human Immunodeficiency Viruses (HIV) coinfections, UK: Patients want support to address social barriers, and Spain: Increased awareness of HCV, HCV treatment and alcohol use.

**Table 2:** Example of Analysis Process

Interview text unit	Codes applied	Codes summary
*“It’s just, you’re on top of it in here, where outside your doing other things, you mightn’t have time to go to hospital, do you know what I mean?.... I know that seems silly but that’s the way it is when you’re on drugs and all, do you know what I mean?... You haven’t time to be going to the hospital.”* (IE-30)	Prison positive, HCV treatment, complex lifestyle, cycle of addiction, competing priorities, barrier to treatment, LTFU	Prison as a haven for treatment
*“What did piss me off about that first doctor when I went to xx, because that could put a lot of people off a lot worse than me. And yet I honestly feel he was trying to sort of arse poke me, getting a reaction it was getting worse. Whereas I think he said, “don’t give him an appointment before November”, or something along them lines. I don’t know. Someone else wouldn’t have gone back. What was it he said? Someone else. I think his exact words were “well we’re not going to treat people who, I forget the exact words, that’s still using or. Yea I said you’re not going to get anyone turning up for treatment. And I got the impression he thought “good” … So, yea I really got the impression he didn’t want to. And I had to change my whole life before he’d even consider treating me.”* (UK-3)	Judgement, barrier to treatment, HCP experience, motivation, appointment, barrier to treatment, LTFU, cycle of addiction, IDU stigma, barrier to treatment, HCP experience, judgement, barrier to treatment, personal motivation, competing priorities, LTFU	Lost to follow up
*“Due to the problems faced by the Romanian health care system it was difficult for me to be tested for HCV viral load or liver fibrosis staging in the hospital. Due to enrolment in HepCare Project I could benefit of these investigations and finally initiated the treatment. Without this program, probably I would have got treatment but much later, at an advanced liver disease stage and with a higher risk of complications”* (RO)	Barriers to treatment, eligibility requirements, delayed treatment, LTFU, HepCare, HCV support, improved treatment access, offered treatment, delayed treatment, advanced disease	HepCare improved access to treatment
*I: Would you have received treatment if it wasn’t for this program?* *P: I don’t know, probably no because I didn’t know about the new treatment. I thought that the treatment was the interferon and I said one day that I wouldn’t ever be treated with this”* (SP- 5)	Treatment regime, new treatment unknown, HCV misinformed, HepCare, HCV educated, awareness, access to services	HCV education needed

**Table 3:** Theme Progression

First round codes	Categories	Themes
Not know treatment was available, Not know there is new treatment, Not assisted in accessing treatment, diagnosed but not offered treatment	Lack of agency	Lost to Follow Up
treatment unavailable, strict eligibility requirements, cost, stigma, peer v HCP, waiting times, mistrust HCP	Barrier to accessing treatment	
Homelessness, mobility, instability, complex lifestyles, cycle of addiction, mental health, HCV not priority, family problems, regret, IDU history, IDU	competing priorities	
concerns about treatment, transmission fears, disclosure, HCV stigma, shame, burden, depression, HCV denial, want support, isolation	HCV fears	
Never offered treatment, not access services, dislike hospital, HIV > HCV, background	HCV status unknown	HepCare improved access to treatment
Prison, appointments, resolve, want treatment, want support, HCV support, HCP experience, no judgement, new opportunities. HepCare vouchers	Access treatment	
Adherence, motivation, treatment side effects, trust HCP, HCV symptoms, outreach services, addiction services, personal relationships	treatment experience	
HCV free, positive outlook, new opportunities, self-care, mental health, resolve, personal motivation	HCV free	
Alcohol use, sharing gear, transmission fears, confidence in treatment, risk reinfection, HCV misinformed, HCV peer discussion	awareness	HCV education

## Results

The first part of this section addresses common themes between all four countries enabling a clear insight into shared obstacles around Hepatitis C treatment and linkage to care. Following on from this, site-specific themes are then discussed to underpin the importance of implementing tailored interventions to meet community demands within all four countries.

### Common themes

#### Hepatitis C patients lost to follow up

While reasons for patients lost to follow up was not homogenous between the sites, there were similar threads as to why patients were not linked to care. In this section participants share their perceptions, by firstly indicating that stigma remains a barrier, particularly with PWIDs, for accessing treatment. Patients with a history of drug injection, feel stigmatised when they are judged for their past and are solely labelled as drug users [21]. Patients also identified concerns about treatment, difficulty accessing appointments and competing priorities as additional barriers to treatment. Interviews highlighted different underlying components between sites, reiterating the need for site-specific tailored interventions. In Ireland, patient mobility was often pressurized by homelessness, the cycle of addiction and disadvantaged backgrounds. Many patients referred to traumatic family events as a catalyst for returning to a cycle of drug abuse resulting in their reluctancy to access care:

*“And that brought back memories because I lost me ma and da and me other brother. Me brother was found above Christchurch OD’ed as well, you know, so it just brought back memories, and I drifted back down, and I start taking crack as I was selling gear, and then I start selling crack at night, only getting half an hours sleep for months, and I got caught up with all the shit and I got locked up you know. This [prison] saved me if I’m being honest. So now, now my mind is set not to go back there.”*

*“yeah, they asked me about it [treatment] once or twice but I was, to be honest with you doctor, my life was chaotic, I couldn’t manage it, do you know what I mean?”*

In Romania, most patients were ineligible for treatment at the time of diagnosis, noting that they were not advanced enough to be referred for treatment and were advised to come back when their HCV condition had worsened:

*“Even if I asked for it [treatment] each time I had the occasion, I never received it. I insisted on receiving the treatment when I heard about DAA treatment for the first time … I really wanted to cure my HCV and I came to the hospital more times to talk to my doctor about it. But she explained me that she can’t help me because unfortunately the treatment wasn’t available for everyone in Romania. Only patients with advanced liver fibrosis could benefit of it.”*

In the UK, patients were diagnosed and informed of the need for treatment but are not assisted in obtaining specialist referrals or further appointments. With competing priorities and complex lifestyles, patient noted the need for assistance in being linked to care:

*“They just told me I had it and had to go to the doctor's and I never chased that up do you know what I mean?”*

Similarly, in Spain many patients were not offered treatment following their diagnosis, with some forgetting their diagnosis until the HepCare program retested them and linked them to care:

*“Somebody told me the virus was sleeping so I forgot until you came to our centre.”*

#### HepCare improved access to treatment

HepCare Europe used a variety of interventions across the four sites to ensure access to HCV testing and retention in HCV care. In Ireland, providing access to treatment in the prison system was key to engaging and retaining patients in treatment. The patients identified prison as not only an acceptable setting, but also as an ideal setting in which to receive treatment, as the structure of life in prison provides stability, helps with adherence to treatment and is associated with improved physical and mental health:

*“It’s just, you’re on top of it in here [prison], where outside your doing other things, you mightn’t have time to go to hospital, do you know what I mean?.... I know that seems silly but that’s the way it is when you’re on drugs and all, do you know what I mean?... You haven’t time to be going to the hospital.”*

*“[about receiving treatment in the prison] yeah, because when you are outside like, you know, people have things to get down on, especially if you are taking drugs then you kinda forget what you’re doing, so being in here and getting in here, it’s a lot better because sort of follow a, you’re not going around taking drugs and the next day forgetting to take your medications.”*

Patients in Romania asserted personal responsibility for their HCV treatment, with many stating that they were motivated and wanted treatment. They noted that the HepCare Europe programme helped them to access treatment far faster than they would have, if left to secure it themselves. Patients noted that HepCare removed some of the barriers to treatment, such as securing appointments with specialists and helped them remember appointments:

“*But for the Project [HepCare], the treatment would have been delayed for sure. Probably I should have waited a few more years till somebody would have thought about me, would have remembered that I am also HCV positive and would have initiated treatment.”*

*“Due to enrolment in HepCare Project I could benefit of these investigations and finally initiated the treatment. Without this program, probably I would have got treatment but much later, at an advanced liver disease stage and with a higher risk of complications.”*

In the UK, HepCare vouchers were an important incentive for engaging patients and keeping them linked to care. Many patients mentioned that the vouchers for bus fares and for meals were particularly helpful in improving attendance and keeping patients involved in the treatment process:

*“You know what made me go in that van? You wanna know the truth … because I was getting a bag of crisps and a bar of chocolate free … They saved my life. All for a bar of chocolate and crisps.”*

Nine out of ten patients in Spain felt that HepCare made accessing treatment easier through testing and making patients aware of their infection, improving access to services and decreasing the waiting times for appointments:

*“In only three months I started treatment and with the waiting lists in Spain, I’m sure I would have had to wait longer [without HepCare].”*

HepCare was also crucial in helping patients stay on treatment through reminders for appointments. These interventions combined with their personal motivation were key to achieving SVR:

*“It [HepCare] has been very helpful to us. They arranged the appointments and reminded me when I had to go to the specialist service.”*

#### Need improved HCV education

Underlying the main themes, was the steady mention of both misinformation and outdated information on HCV effects, treatment and transmission; all of which contribute to stigma (judgement) when being treated for HCV, transmission and fear. Patients identified a widespread need for education, identifying that they themselves, the public and healthcare providers would benefit:

*“That’s the whole reason why I’m doing the interview- to help some other people. ‘Hey, this isn’t as bad as what people make it out to be’ and encourage them to change their life around, just need the right tools.”*

In Ireland, the prisoners discussed stigma towards those who have HCV and those with a history of injected drug use who need treatment, as a result of a lack of education:

*“It’s [stigma] just like, I suppose, ‘don’t be around him’, ‘you’d catch something off him’, you know. ‘He’s dirty’, you know, that kind of way.”*

*“There should be more education in the prisons about it [HCV], for people who don’t need it, you know what I mean; because there is a stigma to it, you know what I mean. There’s a stigma to someone on gear, but there is a bigger stigma to someone on gear that used needles.”*

Additionally, patients still associate the dangers and difficulties of interferon treatment and liver biopsies with HCV treatment, despite the arrival of new, less invasive DAA therapies:

*“So, she died of hepatitis, doctor. She went in to get her spleen, she got her spleen took out, and she was drinking at this point, and they told her that the way her liver has gone so far now, you have 6 months to live, we give you 6 months... And they says if you do this treatment, if must have been the interferon— It reacted wrong on her body. The next day after taking it she swelled out. She died within two or three days; you know. so that was always in my mind, you know.”*

In some instances, patients were not aware that there was any treatment available for HCV at all:

*“Like I said, mate I didn’t know there was like this treatment until I was in … . … . In rehab. Before that I didn’t know you could get treatment to get rid of it.”*

“*At the hospital they confirmed me that I am HCV positive and gave me treatment for HIV. Only recently when I was enrolled in HepCare program I was told about HCV treatment.”*

In Romania, many people had been previously told or were aware of the strict treatment criteria, which required advanced disease in order to receive treatment. HepCare made patients aware that the eligibility requirements for treatment had changed allowing them to avail of treatment and linking them to care.

When receiving treatment, patients who are part of the HepCare Europe programme are educated on HCV and transmission risks to allow them to feel engaged in their treatment process and to take better precautions going forward. Despite this, the interviews indicated that there are some areas that need extra attention, such as providing a service that accommodates various literacy levels. Patients in the UK highlighted the need for staff to slow down and take more time with patients to help them understand HCV risk factors and effects:

*“Simplifying terms because a lot of people there can't read and write. So will you show the diagrams cos they weren't, for example, because they see the diagrams right here right now of the liver is already and everything the red level of whatever love of blah, blah, blah is proper healthy, this one yeah. Cos I might not be able to read the, the bullet points on the left-hand side. ‘that grey area that is scarring’ and that, next picture and all that grey it is cirrhosis, sort of explain it like that.”*

There was also confusion about the risk of reinfection:

*“We can’t get it [HCV] though. If we get reinfected, we can’t get it [HCV] again.”*

From this, patients suggest the need for HCV education along the entire cascade of care to offer correct knowledge about HCV and the risks of re-infection.

### Site specific findings

#### IRELAND: achieving SVR is associated with new opportunities

Once they achieved SVR, patients expressed resolve and motivation to stay drug-free and to not place themselves at risk of reinfection. While Ireland was not the only site that alluded to this, it was far more pronounced in Ireland, potentially because achieving SVR was discussed in light of leaving incarceration. Patients expressed positivity and excitement for new opportunities on release, with many patients securing future plans:

*“When I’m going to leave the prison now, when I do that, when I done that course of tablets, it was like getting the second chance in life to me, you know what I mean. So, when I am leaving prison now, I am going to a place called [Drug treatment service] for two years. I’m going there when I leave prison, and then I am going over to Norway. When I complete [Drug treatment service] I can volunteer in another [Drug treatment service] in Norway, so that’s my plan, doctor. You know what I mean.”*

#### ROMANIA: HCV is comparatively less important in light of HIV Coinfections

With 8 out of 10 interviewees coinfected with HIV, an HIV diagnosis was often the stimulus for attending hospital and taking medication regularly. For many previously diagnosed with HCV, no further action to address their HCV infection was taken until after an HIV diagnosis, which was attributed both to unavailability of HCV treatment and a lack of HCV symptoms resulting in a lack of urgency:

*“I was, but I didn’t care too much about it … I didn’t attend the medical appointments till 2012 I guess, when I was diagnosed with HIV.”*

#### UK: patients require support to conquer personal and social barriers

Many patients expressed a desire for treatment and a need for support in addressing HCV and associated problems in their lives that stop them from accessing treatment. Patients want support in getting off of methadone treatment, with many noting that “you are not fully clean” until you get off of OST. Patients admitted to missing appointments, being impatient at times and having difficulty focusing on directions and remembering instructions yet asked that the staff not give up on them and help them work through these issues:

*“Yeah, I missed 3 appointments and it was my own fault for that so I missed 3 appointments with her Jenny, that was her name, it was just the welfare, so she said that she can't give me another appointment. But I really need her to give me another appointment [long pause] because when you get stuck in a rut, you get stuck in a rut.”*

*“don't give up on people, and that's the main thing.”*

#### SPAIN: increased awareness of HCV, treatment and alcohol use

Patients identified that they were happy to have increased awareness of HCV treatment and transmission methods, as well as a better understanding of the effects of alcohol use. While patients may have been previously diagnosed, many did not understand that they were still infected, and were happy to be retested and then treated:

*“The program has made me aware of the two problems I had in my life: hepatitis c and alcohol.”*

*Summary of quotes*

**Table:** 

Categorical Areas	Examples of Quotes
HCV stigma	*“I am very happy I am cured. My family can be proud of me now … I can give them a new life, the life they desired. My son won’t know that his father was HCV positive. We can do different activities together when he will grow up. I can hardly wait to play football with him, and I also hope that he will enjoy jogging. My wife won’t be afraid that she will get the virus from me. I can live a normal life from now on.”* (RO)
Barrier to treatment	“*What did piss me off about that first doctor when I went to xx, because that could put a lot of people off a lot worse than me. And yet I honestly feel he was trying to sort of arse poke me, getting a reaction it was getting worse. Whereas I think he said, “don’t give him an appointment before November”, or something along them lines. Em I don’t know. Someone else wouldn’t have gone back. What was it he said? Someone else. I think his exact words were “well we’re not going to treat people who, I forget the exact words, that’s still using or. Yea I said you’re not going to get anyone turning up for treatment. And I got the impression he thought “good” … So, yea I really got the impression he didn’t want to. And I had to change my whole life before he’d even consider treating me.” -UK4*
Competing priorities	*“Because if I was outside, I don’t know if I would be able to, to have the discipline to take new medication every day.” -IE33*
Access to treatment	*“The fact that you got vouchers believe it or not, was one of the reasons, sad as it is., when you first turned up. From then on it gives you a bit more motivation to sort your life out a bit.” -UK4*
Treatment experience	*“Brilliant, yeah, very positive. Because if I was outside, I don’t know if I would be able to, to have the discipline to take new medication every day. And especially in Pats, the medics over there and the nurses over there are great and everything, you know what I mean. They are brilliant, they are very good.” -IE33*
Awareness	*“didn’t know, I had no, I had no … they say knowledge is power. I didn’t know. If I had known that you could caught it like that, then I wouldn’t have”-IE36*
Lack of agency	*“At first I didn’t know about the infection. Before 2013 I had no sign or symptom of diseases, so I didn’t know I need it. Then, since 2013, I was in active evidence at the hospital for my HIV infection and I came here regularly for treatment and investigations, but nobody told me about treatment. I think it wasn’t available till recently.” -RO10*
HCV free	“*And generally, it [treatment] has made me, it gives you like that one thing. That one thing that you’re trying to change your life a bit. So even just that one thing. By sorting that out you’re heading in the right direction and it makes you not want to catch it again for sure. Yea, there’s a lot of positives out of it.”* -UK3

## Discussion

This study confirmed many well documented barriers to treatment, as well as demonstrating how innovative and flexible interventions have the ability to improve patient engagement and retention in care.

### HepCare Europe interventions address barriers to treatment

The barriers to HCV testing and treatment for People Who Inject Drugs (PWID) are well documented in literature [15, 16, 18, 19, 22]. This study confirmed many of the identified barriers vulnerable populations face when accessing HCV treatment including stigma, concerns about interferon treatment, competing priorities, mistrust between healthcare providers and PWID, and difficulty accessing and navigating the treatment pathway. This study, however, is in the unique position to understand how these barriers compare and contrast across European countries with different populations, allowing for a better understanding of the acceptability of the tailored interventions.

### Need improved HCV education

Across all sites, the need for improved and widespread education on HCV was evident. Not only in order to combat stigma and decrease transmission rates, but also to engage patients in treatment. Similar to recent studies [12, 23], patients in HepCare Europe also expressed a lack of knowledge and scepticism of the new DAA therapy, viewing it as risky or uncertain. HepCare patients echoed fears of interferon treatments, recalling ‘horror stories’ of friends and family, with interviews demonstrating a continued misunderstanding of HCV, with patients confused about transmission, reinfection and new treatment options. Many patients discussed their own misunderstandings as a driving force to want to engage in peer support work themselves, wanting to let others know about the improved DAA therapy and opportunities that come with it.

#### Comparison with other literature

Current literature notes that competing priorities and the deprioritization of HCV discourage patient engagement [12, 24]. As was suggested previously [17, 22], setting services within the prison system is essential for engagement and adherence with the Irish cohort. Patients identified that they would be unlikely to complete treatment outside of prison services and found prison to be an ideal setting to receive treatment due to their improved mental health and stability.

In the UK cohort, patients expressed the need for assistance in being linked to care, specifically with making specialist appointments. HepCare patients noted that they received no support in making appointments for treatment after diagnosis, reiterated findings from Rhodes et al. [23] that patients in London are not empowered to demand or access treatment.

In Romania, 8 out of 10 patients interviewed were coinfected with HIV. Rhodes et al. has attributed co-infection to the deprioritization of HCV [23] and could also explain why Romanian patients did not highlight stigma as an issue as is suggested in other studies [13]. Coupled with coinfection, many Romanian patients were previously turned away from treatment as they failed to meet eligibility criteria.

In Romania, DAA therapy was only available for patients with advanced liver fibrosis (Metavir F3 and F4 score) until 2018 [20], which was understood and reiterated by the patients.

Judgement from healthcare providers, both surrounding HCV and their injecting drug use, led to patients delaying treatment in both Romania and the UK. Treloar et al. [16] suggests that judgement contributes to patients not considering themselves as legitimate patients. HepCare confirmed that the use of peer workers and the relationships between healthcare providers and patients is crucial for the uptake of services and adherence to treatment [12].

HepCare Europe demonstrated a model for improved HCV testing and linkage to care for vulnerable populations. The use of innovative interventions to engage patients, presenting testing and treatment opportunities outside of hospitals in areas that the patients felt more comfortable has proven acceptable to patients.

### Strengths and limitations

This is the first multi-country qualitative research study on DAA therapy interventions. The study was unable to achieve the desired recruitment criteria in some cohorts due to factors beyond control, such as: patients left treatment (prison), patients unwilling to participate, or patients not having completed treatment yet. The addition of focus groups with healthcare providers could provide useful additional information on the issues encountered with these interventions.

### Implications for practice, policy and future research

This study demonstrates the ability of targeted interventions to improve the identification, evaluation and treatment of HCV in vulnerable populations. Through a unique system of care involving support in linkage to care and intensified peer support, HepCare Europe demonstrates the importance of investing in personalized medical care for vulnerable populations. The ability of HepCare to not only enrol patients, but to keep them engaged in care, demonstrates the need for services to be moved out of the hospital and into the communities of these populations, where they face less stigma and can be incentivized to present for testing and treatment.

## Conclusions

This is the first multi-country qualitative study on perceptions of DAA therapy. The unique cohort allows us to compare and contrast treatment modalities and acceptability across Europe, identifying common themes and highlighting areas for customization. Patients in all sites found HepCare to improve their HCV treatment experience through decreased waiting times, assistance navigating the care pathway and providing services in non-traditional locations which patients found more suitable to their complex lifestyles. HepCare Europe represents a best-practice example for treatment delivery with site specific modifications in key populations.

## Data Availability

The data that support the findings of this study are available from the corresponding author, John S. Lambert upon reasonable request.

## Abbreviations

HCV: Hepatitis C Virus
EU: European Union
WHO: World Health Organization
PWID: People Who Inject Drugs
LTFU: Lost To Follow Up
UK: United Kingdom
DAA: Direct Acting Antiviral
SVR: Sustained Virologic Response
HIV: Human Immunodeficiency Viruses

## References

[1] Perz JF, Armstrong GL, Farrington LA, Hutin YJF, Bell BP (2006). The contributions of hepatitis B virus and hepatitis C virus infections to cirrhosis and primary liver cancer worldwide. J Hepatol.

[2] World Health Organization. Global hepatitis report, 2017. Geneva; 2017. [cited 2018 Apr 30]. Available from: http://apps.who.int/iris/bitstream/handle/10665/255016/9789241565455-eng.pdf;jsessionid=39DE8B60B231C47D1E3BD37BFE9CB595?sequence=1

[3] Chen E, Morgan TR (2006). The natural history of hepatitis C virus (HCV) infection. Int J Med Sci.

[4] World Health Organization (2016). *Global Health Sector Strategy on Viral Hepatitis* 2016–2021.

[5] Hope VD, Eramova I, Capurro D, Donoghoe MC (2014). Prevalence and estimation of hepatitis B and C infections in the WHO European region: a review of data focusing on the countries outside the European Union and the European free trade association. Epidemiol Infect.

[6] Zurhold H, Haasen C, Stöver H (2005). Female drug users in European prisons: a European study of prison policies, prison drug services and the women’s perspectives.

[7] Fazel S, Geddes JR, Kushel M. The health of homeless people in high-income countries: descriptive epidemiology, health consequences, and clinical and policy recommendations. Lancet. 2014. 10.1016/S0140-6736(14)61132-6. 10.1016/S0140-6736(14)61132-6 PMC4520328 25390578

[8] Beijer U, Wolf A, Fazel S (2012). Prevalence of tuberculosis, hepatitis C virus, and HIV in homeless people: a systematic review and meta-analysis. Lancet Infect Dis.

[9] Bajis S, Dore GJ, Hajarizadeh B (2017). Interventions to enhance testing, linkage to care and treatment uptake for hepatitis C virus infection among people who inject drugs: a systematic review. Int J Drug Policy.

[10] Bruggmann P, Grebely J (2015). Prevention, treatment and care of hepatitis C virus infection among people who inject drugs. Int J Drug Policy.

[11] Avramovic G, Oprea C, Surey J, Story A, Macías J, Cullen W (2020). HepCheck: characteristics of the patient population with active infection as defined by HCV RNA. Int J Infect Dis.

[12] Harris M, Bonnington O, Harrison G, Hickman M, Irving W (2018). Understanding hepatitis C intervention success: qualitative findings from the HepCATT study. J Viral Hepat.

[13] Whiteley D, Whittaker A, Elliott L, Cunningham-Burley S (2018). Hepatitis C in a new therapeutic era: Recontextualising the lived experience. J Clin Nurs.

[14] Swan D, Cullen W, Macias J, Oprea C, Story A, Surey J (2018). Hepcare Europe- bridging the gap in the treatment of hepatitis C: study protocol. Expert Rev Gastroenterol Hepatol.

[15] Harris M, Rhodes T (2013). Hepatitis C treatment access and uptake for people who inject drugs: a review mapping the role of social factors. Harm Reduct J.

[16] Treloar C, Rance J, Backmund M (2013). Understanding barriers to hepatitis C virus care and Stigmatizaon from a social perspective. Clin Infect Dis.

[17] Swan D, Long J, Carr O (2010). Barriers to facilitators of hepatitis C testing, management, and treatment among current and former injecting drug users: a qualitative exploration. AIDS Patient Care STDS.

[18] Nguyen O, Dore G, Kaldor J (2007). Recruitment and follow-up of injecting drug users in the setting of early hepatitis C treatment: insights from the ATAHC study. Int J Drug Policy.

[19] Harris M, Ward E, Gore C. Finding the undiagnosed: a qualitative exploration of hepatitis C diagnosis delay in the United Kingdom. J Viral Hepat. 2016;23:479–86. 10.1111/jvh.12513 26924296

[20] HepCare Manifesto: Bridging the gap in the treatment of Hepatitis C. OCT 2018: 1-4. https://www.ucd.ie/medicine/t4media/Hepcare%20Europe_Manifesto%20%20FINAL%2026OCT2018.pdf.

[21] Muncan B, Walters SM, Ezell J, Ompad DC (2020). “They look at us like junkies”: influences of drug use stigma on the healthcare engagement of people who inject drugs in New York city. Harm Reduct J.

[22] Wolfe D, Luhmann N, Harris M (2015). Human rights and access to hepatitis C treatment for people who inject drugs. Int J Drug Policy.

[23] Rhodes T, Harris M, Martin A (2013). Negotiating access to medical treatment and the making of patient citizenship: the case of hepatitis C treatment. Sociol Health Illn.

[24] Jones L, Atkinson A, Bates G (2014). Views and experiences of hepatitis C testing and diagnosis among people who inject drugs: systematic review of qualitative research. Int J Drug Policy.

